# Optimized SPECT Imaging of ^224^Ra α-Particle Therapy by ^212^Pb Photon Emissions

**DOI:** 10.2967/jnumed.122.264455

**Published:** 2023-07

**Authors:** Lars Tore Gyland Mikalsen, Monika Kvassheim, Caroline Stokke

**Affiliations:** 1 Division of Radiology and Nuclear Medicine, Oslo University Hospital, Oslo, Norway;; 2 Department of Life Sciences and Health, Oslo Metropolitan University, Oslo, Norway;; 3 Faculty of Medicine, University of Oslo, Oslo, Norway; and; 4 Department of Physics, University of Oslo, Oslo, Norway

**Keywords:** optimization, SPECT, Ra224, Pb212, α-particle therapy

## Abstract

In preparation for an α-particle therapy trial using 1–7 MBq of ^224^Ra, the feasibility of tomographic SPECT/CT imaging was of interest. The nuclide decays in 6 steps to stable ^208^Pb, with ^212^Pb as the principle photon-emitting nuclide. ^212^Bi and ^208^Tl emit high-energy photons up to 2,615 keV. A phantom study was conducted to determine the optimal acquisition and reconstruction protocol. **Methods:** The spheres of a body phantom were filled with a ^224^Ra-RaCl_2_ solution, and the background compartment was filled with water. Images were acquired on a SPECT/CT system. In addition, 30-min scans were acquired for 80- and 240-keV emissions, using triple-energy windows, with both medium-energy and high-energy collimators. Images were acquired at 90–95 and 29–30 kBq/mL, plus an explorative 3-min acquisition at 20 kBq/mL (using only the optimal protocol). Reconstructions were performed with attenuation correction only, attenuation plus scatter correction, 3 levels of postfiltering, and 24 levels of iterative updates. Acquisitions and reconstructions were compared using the maximum value and signal-to-scatter peak ratio for each sphere. Monte Carlo simulations were performed to examine the contributions of key emissions. **Results:** Secondary photons of the 2,615-keV ^208^Tl emission produced in the collimators make up most of the acquired energy spectrum, as revealed by Monte Carlo simulations, with only a small fraction (3%–6%) of photons in each window providing useful information for imaging. Still, decent image quality is possible at 30 kBq/mL, and nuclide concentrations are imageable down to approximately 2–5 kBq/mL. The overall best results were obtained with the 240-keV window, medium-energy collimator, attenuation and scatter correction, 30 iterations and 2 subsets, and a 12-mm gaussian postprocessing filter. However, all combinations of the applied collimators and energy windows were capable of producing adequate results, even though some failed to reconstruct the 2 smallest spheres. **Conclusion:** SPECT/CT imaging of ^224^Ra in equilibrium with daughters is possible, with sufficient image quality to provide clinical utility for the current trial of intraperitoneally administrated activity. A systematic scheme for optimization was designed to select acquisition and reconstruction settings.

## Visual Abstract

**Figure:**
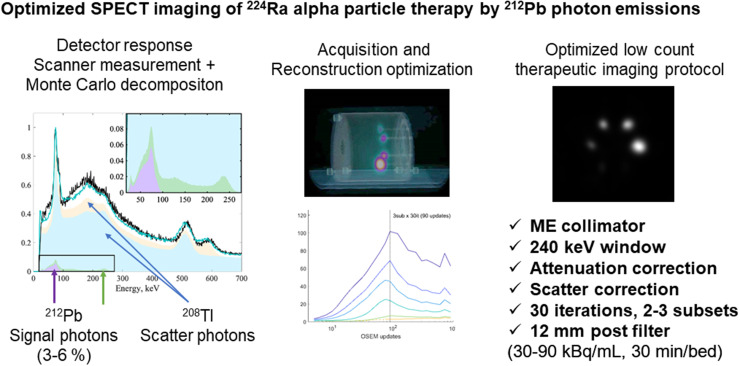

Targeted α-particle therapy has seen increasing interest during the last decade (1). α-particles are densely ionizing with short-range and high-energy (HE) transfer. Imaging of the patient-specific uptake is commonly considered difficult, because only small amounts of activity are used. Still, a few studies have investigated imaging of different nuclides and found that relevant clinical information can be obtained. Planar imaging has been performed, and although ^223^Ra-RaCl_2_ (Xofigo; Bayer) is the most extensively studied (2*,*^3^), other α-particle therapeutics have also been investigated by imaging (4). SPECT imaging has rarely been performed, although images of decent quality have been obtained on a few occasions, for example, in a trial using a ^213^Bi-labeled substance for therapy of gliomas (5) and for ^225^Ac-PSMA (6). ^149^Tb is in a special position among α-emitters, because it also emits positrons and allows PET imaging (7).

^224^Ra adsorbed to calcium carbonate microparticles is being investigated for intraperitoneal therapeutic use in 2 phase 1 trials. The nuclide has a half-life of 3.63 d and emits 4 α-particles as it decays to stable ^208^Pb (8). The shorter half-life of ^224^Ra than of ^223^Ra is considered an advantage for this treatment (9). X-ray and medium-energy (ME) γ-emissions, predominantly from ^212^Pb, can facilitate imaging (Table 1). In the trials, activity will be escalated from 1 to 7 MBq, a range quite typical for α-emitter therapy. The relatively small intraperitoneal distribution volume contributes to expectations of increased concentrations at these activity levels, relative to systemic therapies, and a corresponding minimal background outside the intraperitoneal cavity. In addition to low amounts of activity providing little signal, imaging is complicated by the high background of scattered photons. Hence, a highly optimized imaging protocol is necessary. Although an earlier study that performed planar imaging of 3 patients with intraperitoneal ^212^Pb-TCMC-trastuzumab (10) gave an idea of the possibility for imaging, we have been unable to find previous imaging studies for ^224^Ra.

**TABLE 1.** ^224^Ra Decay Chain Photon Emissions (Cutoff, 40 keV and 0.5%)

Nuclide	Energy	*P* (%)	Ray type
^208^Tl, α	73.0	0.8	x
^212^Pb	75.1	10.6	x
^208^Tl, α	75.3	1.4	x
**^212^Pb**	**77.4**	**17.7**	x
^212^Pb	87.1	2.0	x
^212^Pb	87.7	3.8	x
^212^Pb	90.2	0.9	x
^212^Pb	115	0.6	γ
**^212^Pb**	**239**	**43.3**	γ
^224^Ra	241	4.1	γ
^208^Tl, α	277	2.3	γ
^212^Pb	300	3.3	γ
^208^Tl, α	511	8.1	γ
^208^Tl, α	583	30.3	γ
^212^Bi, β	727	4.2	γ
^208^Tl, α	763	0.7	γ
^212^Bi, β	785	0.7	γ
^208^Tl, α	861	4.5	γ
^212^Bi, β	1621	1.0	γ
^208^Tl, α	2615	35.6	γ

*P* = probability of emission per ^224^Ra decay obtained from International Commission on Radiological Protection publication 107 (8); β = β-decay branch of ^212^Bi, 64%; α = α-decay branch of ^212^Bi, 36%.

Value of highest-frequency photon emission, in bold, within each window was used for AC.

The primary objective of the present work was to determine whether SPECT/CT imaging is meaningful under the conditions for the current clinical trial and to identify the optimal imaging protocol. To this end, we aimed to develop a general optimizing routine to maximize the contrast of phantom spheres relative to scatter-induced noise and compared each evaluated acquisition protocol using individually optimized reconstruction settings.

## MATERIALS AND METHODS

### Image Acquisition and Energy Spectra Measurements

A NEMA IEC PET Body Phantom Set (Capintec) was applied without the lung cylinder insert, using a ^224^Ra-RaCl_2_ solution in the spheres and nonradioactive water in the background chamber. ^224^Ra was produced with a generator based on the parental nuclide ^228^Th (9). The phantom contained 6 spheres with diameters of 10, 13, 17, 22, 28, and 37 mm and volumes of 0.52, 1.15, 2.57, 5.57, 11.49, and 26.52 mL, for a total of 47.82 mL. The spheres were filled with ^224^Ra-RaCl_2_ solution containing ethylenediaminetetraacetic acid and saline water. Imaging was performed at ^224^Ra concentrations of 90–95 kBq/mL (4.4–4.6 MBq total) and 29–30 kBq/mL (1.4 MBq total) in the spheres, denoted as 90 and 30 kBq/mL, respectively. Acquisitions were made on a Symbia Intevo Bold system (Siemens) with a 1-cm (3/8 inch) crystal size using ME and HE collimators, a 20% energy window centered at 240 keV, and a 40% window centered at 80 keV, with dual scatter windows of 5% and 20%, respectively (Table 2). A single bed position was acquired using a 256 × 256 matrix, noncircular orbit, acquisition during steps, and 60 views of 30 s each. CT was acquired at 130 kVp with 2.49 mGy CTDIvol and reconstructed with a soft filter (B08). Quality-assurance procedures were followed according to manufacturer recommendations.

**TABLE 2.** SPECT Acquisition Windows

Candidate imaging window	Lower scatter window	Emission window	Upper scatter window	AC	ē	∑*P* (%)
X-ray window, 80 keV	48-64	64-96	96-112	77.4	79.2	43
ME γ-window, 240 keV	204-216	216-264	264-276	239	239	48

ē = theoretic average energy of primary emissions within window; **∑***P* = Total probability of emission within the energy window per ^224^Ra decay.

In addition, a single acquisition was performed with ME collimators, 3 s/view, and the 240-keV triple-energy window. The phantom spheres contained 20 kBq/mL at the time of imaging. Because of the short acquisition time, the dataset is considered equivalent to 2 kBq/mL using the standard protocol of 30 min. Energy spectra were acquired with and without the water-filled phantom hull and with and without collimators mounted.

### Monte Carlo Simulations of Energy Spectra

To gain better insight into the composition of energy spectra, and the nature of the observed high scatter, Monte Carlo simulations were performed using the Geant4 Application for Tomographic Emission (version 9.0; OpenGATE Collaboration) (11*,*^12^). The SPECT detectors were modeled with collimators, crystals, back compartments, and shielding. The back compartments were described in detail, with lightguides, electronics, and photomultiplier tubes, to accurately model the backscatter of HE photons (13*,*^14^). The source was simulated using a UserSpectrum, including all photon emissions from the decay chain with emission probabilities larger than 0.1% per ^224^Ra decay. Energy blurring was modeled using a linear law with 13% resolution at 80 keV and a slope of −0.091/MeV after calibration with the experimental spectra. The electromagnetic option 4 physics list was used. The obtained spectra were validated against experimental results with ME collimators, HE collimators, and no collimators. After validation, the simulations were repeated including only selected emissions from the ^224^Ra decay chain; the γ-emission of 2,615 keV, γ-emission of 583 keV, x-ray emissions of 70–90 keV, and γ-emissions of 239 and 241 keV were simulated separately.

### Reconstructions

Images were reconstructed in Syngo MI Applications, version VB20B (Siemens Healthcare and Toshiba Medical Systems Corp.), using Flash3d. Images were reconstructed with and without scatter correction (SC) at 24 levels of iterative updates (iteration × subsets), ranging from 5 to 900 total updates (Supplemental Table 1 [supplemental materials are available at http://jnm.snmjournals.org]); iterations were preferentially increased over subsets. CT-based attenuation correction (AC) was used. Triple-energy windows were used for SC (Table 2).

Next, 9-, 12-, and 16-mm gaussian filters were used for postfiltering (AC and SC+AC), and SC input data were prefiltered using 9-, 16-, and 20-mm gaussian kernels, respectively, for increasing postprocessing filter strength. The reconstructed matrix was 256 × 256 × 164, with isotropic 2.40-mm voxels. In total, 1,296 reconstructions were performed, 144 per acquisition. Images are shown on a linear gray scale from zero to the maximum intensity value.

### Image Analysis

Analysis was performed semiautomatically using MATLAB, version 2017 (MathWorks, Inc.). Sphere positions were found on CT images. For each reconstruction, spheric volumes of interest (VOIs) approximating the physical sphere volumes were placed optimally on the SPECT images within 20 mm of the CT position by maximizing the total intensity enclosed. When there was no local maximum, the CT-derived position was used. For each spheric VOI in each reconstruction, the maximum and mean were extracted and normalized by activity and scan duration. A 2.8-L large background VOI was constructed containing the water-filled phantom hull with 40.2-mm margins to spheres and a 26.8-mm margin to the phantom hull. Scatter-induced noise in the background compartment was assessed by peak measurements (largest mean of a fixed-size spheric VOI across all locations in the background VOI) using peak VOI sizes matching each spheric VOI. Signal-to-scatter peak ratio (SSR) was defined as the mean sphere value divided by the volume-matched background peak value.

### Iterative Reconstruction Optimization

The optimal number of iterative updates was defined as the reconstruction that maximized the combined normalized SSR. The SSRs of each sphere, as a function of iterative updates, were smoothed using a 3-element floating average, normalized against the maximum value, and added together, omitting spheres without local maxima.

### Acquisition and Reconstruction Comparisons

The normalized maximum values for the largest sphere, and the combined normalized SSR results for each combination of collimator, energy window, and SC, were compared at the individually obtained optimal number of iterations.

## RESULTS

### Energy Spectra and Contributions from Different Emissions

**FIGURE 1.**
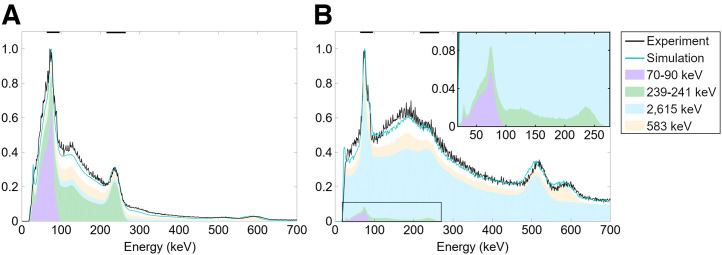
Captured (experiment) and Monte Carlo–simulated (cyan line) normalized energy spectra without (A) and with (B) ME collimator. Energy windows are marked with black lines on top axis. Stacked areas show contribution of imageable x-ray (70–90 keV) and γ-ray (239–241 keV) emissions, as well as scattered photons produced by 2,615-keV and 583-keV emissions. Presence of collimators greatly increased scatter; only low percentage of captured counts are from primary emissions. Supplemental Figure 1 shows for further panels.

Energy spectra (Fig. 1) acquired without collimators showed prominent peaks in the x-ray region (70–90 keV) and around 239 keV and minor peaks near 115, 511, and 583 keV. With collimators, a high background of photons was observed throughout the spectrum, obscuring the 239-keV peak. The Monte Carlo–simulated spectra matched the experimental spectra with reasonable visual agreement (Fig. 1; Supplemental Fig. 1). The results show that the collimator-induced background can be almost entirely explained by secondary photons—from pair production, Compton scatter, and x-ray production in lead—resulting from the 2,615-keV ^208^Tl emission. This accounts for most of the counts in the energy windows as well.

In the acquired image raw data, the total counts were 4.6–10 megacounts (Supplemental Table 2). They were 16% higher in the 240-keV window than in the 80-keV window for both collimators. The ME collimator had 79% more counts than the HE collimator. The triple-energy window estimates of scatter ratios underestimated scatter according to Monte Carlo results. The estimates showed 66% in the 80-keV window for both collimators and 91% and 89% in the 240-keV window for ME and HE collimators, respectively. However, the Monte Carlo simulations estimated that only 4% and 6% of counts in the 80-keV window with ME and HE collimators, respectively, and 3% of counts in the 240-keV window with either collimator originated from the desired radionuclide emissions. The remaining detections (94%–97%) in each window were secondary photons of higher-energy emissions.

### Optimal Iteration Levels

**FIGURE 2.**
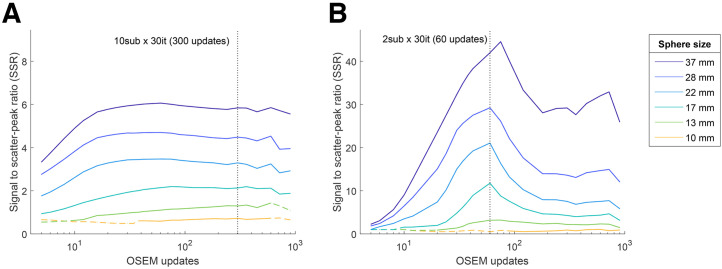
SSR as function of ordered-subset expectation maximization updates (logarithmic scale) for each phantom sphere for selected dataset (ME collimator, 240-keV window, and 12-mm gaussian filter), without (A) and with (B) SC. Dashed segments signify reconstructions where spheres did not show local intensity maximum. Setting that maximized total normalized SSR of all spheres was considered optimal (dashed vertical line). Supplemental Figures 4–7 show further panels.

**FIGURE 3.**
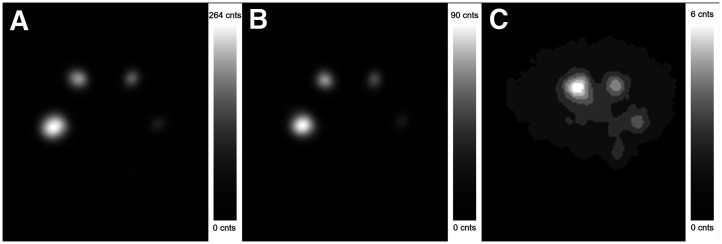
Example of image quality. (A–C) Transaxial slices at each exposure level: 90 kBq/mL (A), 30 kBq/mL (B), and 2 kBq/mL equivalent scan (C), captured using ME collimator and 240-keV window and reconstructed using SC and 12-mm gaussian filter at individually optimal number of iterations. (C) Spheres’ positions are rotated 60° clockwise.

The optimal iterative levels for each combination of collimator, energy window, AC or SC+AC correction, and filters are listed in Table 3. Examples of SSR as a function of iterative updates, used to determine the optimal iterative levels, are shown in Figure 2. Overall, more iterations were tolerated when reconstructing without SC, with a stronger postprocessing filter, or with higher ^224^Ra concentrations. Optimal iterative updates with respect to SSR were 42–900 for AC-only reconstructions and 20–360 for SC+AC. Transaxial slices for a selected protocol at the optimal iterative level are shown in Figure 3. SSR curves for all acquisitions and further image examples are shown in Supplemental Figs. 2–10.

**TABLE 3.** Number of Ordered-Subset Expectation Maximization Updates in Individually Optimized Reconstructions

Parameter	Gaussian filter (mm)	No. of OSEM updates for maximized SSR								ME240 (20 kBq/mL), 3-min scan
		HE80 (95 kBq/mL)	HE240 (94 kBq/mL)	ME80 (90 kBq/mL)	ME240 (90 kBq/mL)	HE80 (30 kBq/mL)	HE240 (29 kBq/mL)	ME80 (29 kBq/mL)	ME240 (29 kBq/mL)	
AC	9	75	90	120	180	42	48	180	90	12
	12	900	120	240	720	42	90	900	300	12
	16	900	600	900	600	120	120	900	300	16
SC+AC	9	90	42	42	75	34	75	120	360	12
	12	120	30	42	90	20	90	120	60	12
	16	180	34	120	120	24	180	120	42	12

OSEM = ordered-subsets expectation maximization; HE80 = HE collimator with x-ray window; HE240 = HE collimator with γ-ray window; ME80 = ME collimator with x-ray window; ME240 = ME collimator with γ-ray window.

Table shows optimal number of ordered-subset expectation maximization updates, according to SSR, for each acquisition, as well as ^224^Ra concentration in phantom at time of imaging. Scan time was 30 min where not otherwise stated.

### Reconstructed Sphere Counts

**FIGURE 4.**
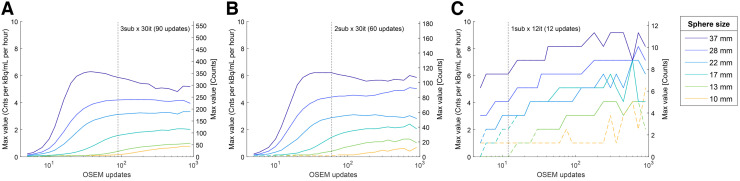
Maximum value in each sphere at each exposure level: 90 kBq/mL (A), 30 kBq/mL (B), and 2 kBq/mL (C) equivalent for selected dataset using ME collimator, 240-keV window, AC, SC, and 12-mm gaussian filter. Exposure-normalized count rates are shown on left axis, and absolute number of counts are shown on right axis. Dashed segments signify reconstructions where spheres did not show local intensity maximum. Supplemental Figures 11–14 show further panels. OSEM = ordered-subsets expectation maximization.

**FIGURE 5.**
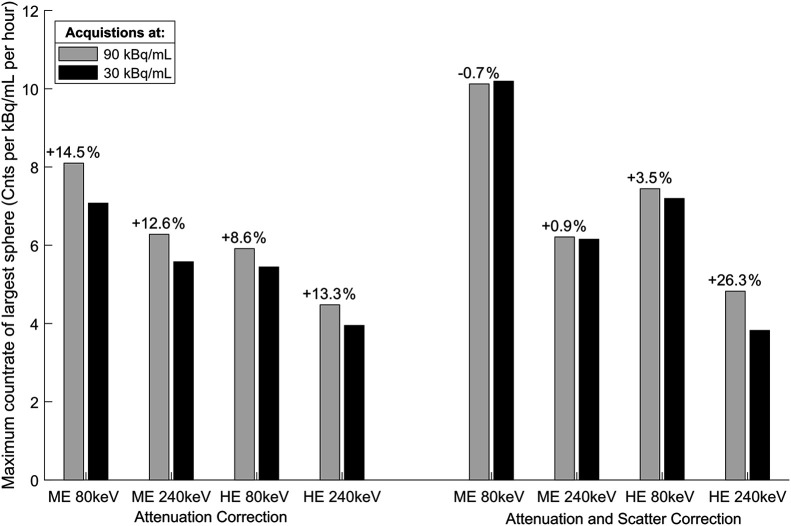
Comparison of maximum voxel count rate in largest sphere at 90 kBq/mL (gray) and 30 kBq/mL (black). Difference in normalized count rates at 90 kBq/mL relative to 30 kBq/mL is shown on top of bars. Acquisitions were reconstructed using optimal settings at 30 kBq/mL for both concentrations.

The maximum values of the spheres reflect a combination of sensitivity and resolution recovery and mostly increased with the number of iterative updates. The larger spheres often reached a plateau, whereas the smaller spheres continued to increase (example in Fig. 4; full results in the supplemental materials). At the SSR-optimized iterative level, we found that the maximum count rate of the largest sphere varied from 3.6 to 10.2 counts/h for 1 kBq/mL depending on window, collimator, and SC (Fig. 5). It was mostly somewhat higher at 90 than 30 kBq/mL; however, near-identical results were obtained with the ME collimator with SC, and consistent results were obtained in the 2 kBq/mL equivalent scan (Fig. 4).

### Acquisition and Reconstruction Comparisons

**FIGURE 6.**
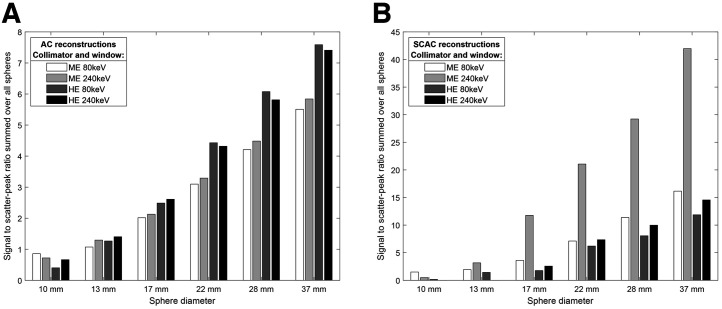
Comparison of maximized aggregated SSR values for each collimator and energy window combination, reconstructed without (A) and with (B) SC. Scatter-corrected images have higher SSRs and are plotted on wider scale. SCAC = Scatter and attenuation corrected.

**FIGURE 7.**
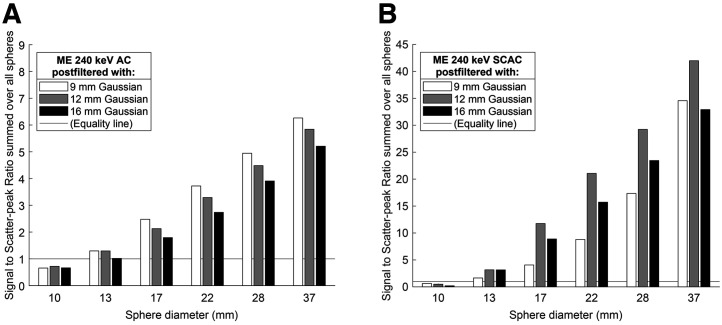
Effect of gaussian postprocessing filter widths on resulting SSRs, for example, setting with ME collimator and 240-keV window, each at individually optimal number of ordered-subset expectation maximization updates. Reconstruction without (A) and with (B) SC. Supplemental Figure 8 shows further panels. SCAC = Scatter and attenuation corrected.

Based on the combined normalized SSR (Fig. 6, and detailed further in the following paragraphs), the optimal acquisition and reconstruction setting for the current investigations was suggested to be ME collimator and 240-keV energy window with SC, AC, and 12-mm gaussian filter. This combination is used for the examples given in Figures 2B, 3, 4, and 7B. Additional figures are available in the supplemental materials, detailing results for all investigated acquisition protocols and reconstruction settings.

#### SC

SC resulted in a higher overall SSR for all collimator and energy window combinations (Fig. 6). However, for some iteration levels, the HE collimator acquisitions failed to reconstruct local maxima for the smallest spheres with SC enabled.

#### Collimator

With AC-only reconstruction, the HE collimator scored higher SSRs than the ME collimator for the 4 largest spheres, and they were equal for the 13-mm sphere. However, the ME collimator outperformed HE for the smallest sphere (Fig. 6). Using SC, the ME collimator had overall better performance in nearly all cases. The ME collimator resulted in higher maximum counts than the HE collimator, when comparing the largest sphere at the optimal iterative update level, for all combinations.

#### Energy Windows

The 2 energy windows gave comparable SSRs, except for scatter-corrected 240-keV images with the ME collimator, which had approximately twice the SSR of the 80-keV window. The 80-keV window provided 29%–80% higher counts than 240 keV, when comparing the largest sphere at the optimal iterative level, as determined by maximized SSR (Fig. 6).

#### Postprocessing filters

Increased postfiltering decreased the maximum counts of both spheres and scatter. In most cases, SSRs decreased as well (Fig. 7), and the best results were obtained with 9-mm postfilters, the smallest examined. The ME-collimated 240-keV window with SC was a notable exception, where the 12-mm filter provided much higher SSR than the 9-mm filter, which gave the poorest result.

## DISCUSSION

We have here established a scheme to optimize independently each combination of collimator, energy window, postprocessing filter, and use of SC for different concentrations of ^224^Ra in equilibrium with daughters, before comparison. Here, the ME collimator with the 240-keV energy window and SC outperformed all other protocols, with SSRs 2–3 times higher than any other combination. Overall, the phantom study shows that tomographic imaging of ^224^Ra in equilibrium with daughters is possible even with low activity. The main contributors to image information are ^212^Pb and daughters; therefore, the findings may be relevant for imaging of ^212^Pb as well.

All 4 combinations of collimators and energy windows were capable of reproducing activity distribution with acceptable accuracy. The maximum number of visible spheres is comparable, but the optimal ordered-subset expectation maximization settings vary, as do the resulting visual texture, contrast, and counts. The most significant factor in the protocol was the use of SC, which was preferable in terms of SSR for all acquisitions, but the ME collimator with the 240-keV window benefited the most (Fig. 6). Overall, we recommend this combination, but the smallest sphere was slightly more visible than with other protocols yet was never clearly defined above the noise floor (SSR < 1). When only HE collimators are available and only the major hot spots are of interest, we suggest using SC. When both weak and strong uptake is important, non–scatter-corrected images may also be produced to visualize the smallest nuclide deposits. Our results suggest that 80- and 240-keV images provide similar SSRs for the HE collimator. Because 80-keV imaging gave higher maximum values, it might be preferable for medium-sized and small patients, but 240 keV is expected to deteriorate less in larger patients. In addition, the scatter triple-energy-window correction appears more accurate for 240 keV, as shown by the Monte Carlo simulations.

In our relevant clinical setting, we expect no or extremely low amounts of activity adjacent to the peritoneal cavity where ^224^Ra therapeutics would be delivered, and no activity was added in the background compartment of the phantom. It can be argued that this is an idealized setup. Still, a high number of counts was reconstructed in the background because of scatter. This led to the definition of SSR to determine image quality instead of more common measures such as signal-to-noise ratio, which often measures noise in a homogeneous background region. SSR is directly interpretable as the contrast ratio between lesions and scatter-induced noise peaks. During optimization, we weighted the SSR of each sphere size equally, which might not be favorable in all clinical circumstances. For specialized uses, conclusions may be drawn from the plotted results (Supplemental Figs. 4–8 and 11–15).

In most cases, SSR was optimized at high iterations, in particular without SC, where several datasets maximized the SSR at 900 updates, the software upper limit (Table 3; Supplemental Figs. 2 and 3). We found that the 9-mm filter (the smallest used) provided the best SSR in nearly all instances, despite high iterations. Our results do not provide a clear recommendation for postprocessing filters but indicate that, depending on the clinical purpose, heavy filtering should be used cautiously. For lower activity, the SSR was typically maximized at lower ordered-subset expectation maximization updates. In particular, the scan equivalent to 2 kBq/mL maximized SSR at only 12 iterations. However, the largest sphere typically achieved convergence after 20–30 iterations, and fewer iterations than this should likely be avoided (Fig. 4; Supplemental Figs. 11–14). Visually, the scan equivalent to 2 kBq/mL also holds up well in this range; encouragingly, the visibility of spheres 3 and 4 increased compared with that of 12 iterations (Supplemental Figs. 9 and 10).

The smallest imageable activity appears to be limited by the resulting desired intensity range of images as the maximum value decreases toward zero. The observed sensitivity values for maximum measurements indicate that to obtain 10 counts in the maximum voxel with a 30-min scan duration, larger lesions need a concentration of about 3–5 kBq/mL and smaller lesions need total activity of about 4 kBq with the suggested protocol. Imaging at the equivalent of 2 kBq/mL confirms that this is achievable, at least for larger spheres. In a therapeutic setting, a steady-state concentration of 4 kBq/mL corresponds to cumulative absorbed doses of up to 8.2 and 0.31 Gy for ^224^Ra and ^212^Pb, respectively, assuming no relocalization (8). Thus, the imageable range encompasses most of the therapeutic range for tumors, although with respect to normal tissues, a negative scan might not be enough to rule out toxicity. The Monte Carlo simulations show that most scattered photons originate in the collimators, not the phantom. This means that activity in the patient easily may contribute to degraded image quality and reduce the visibility of low-concentration deposits, possibly even at large distances. Consequently, the total amount of activity in the patient may be a limiting factor for low-activity detection.

Although phantom studies are a convenient way of determining an imaging protocol before a trial, phantom imaging is highly simplified compared with in vivo patient imaging. We believe that the overall trade-offs of different collimators, energy windows, and SC application will remain similar for this system, but parameters such as iterations and postfilters should be revisited for each clinical context, given the large variation in use for these nuclides. In the context of the 2 phase 1 trials for intraperitoneal administration of ^224^Ra adsorbed in calcium carbonate microparticles, most activity is assumed to be retained within a single bed position without physiologic washout, hence the high starting concentrations in the study design. For the same reason, the study was performed without background in the phantom. Depending on the time of imaging and the location of the VOI—whether a lesion or organs at risk—this needs to be considered, because a blood-pool background or parenchymal uptake will complicate partial-volume effects and reduce visibility, adding the challenge of contrast to that of imaging at low concentrations. The results are most easily interpretable for high-contrast lesions and organs at risk. We have not directly evaluated the possibility of quantitative imaging for dosimetry purposes, but our results indicate that this may be possible (Fig. 5). One should always be aware of the clinical limitations of imaging a selection of nuclides (or a single nuclide) of a decay chain, because this introduces possible relocalization between therapeutic dose deposition and imaging. Although separation of ^227^Th from ^223^Ra has been attempted by spectral decomposition of different windows (15), the relative amount of photons emitted from pre-^212^Pb nuclides in the decay chain of ^224^Ra is probably not sufficient for similar investigations in either window (Tables 1 and 2). Therefore, potential relocalization between ^224^Ra and ^212^Pb will need investigation with supplementary methods (such as blood sampling) in the clinical trials.

## CONCLUSION

The results indicate that low counts are fairly unproblematic for the reconstruction algorithm. The detection limit appears to be in the region of 1–10 kBq/mL with 30-min acquisitions for ^224^Ra and, by extension, ^212^Pb. The HE photons from ^208^Tl produce high amounts of scatter in the collimators, explaining the observed importance of SC. The best results were obtained with the ME collimator.

## DISCLOSURE

No potential conflict of interest relevant to this article was reported.

## Notes

KEY POINTS **QUESTION:** Is clinical SPECT imaging of ^224^Ra- and ^212^Pb-based α-particle therapy possible? **PERTINENT FINDINGS:** The results show meaningful image quality at clinically relevant concentrations. We provide recommendations for an optimized imaging protocol. **IMPLICATIONS FOR PATIENT CARE:** The use of SPECT/CT to verify tumor or normal tissue uptake of α-emitters greatly simplifies imaging endpoints in clinical studies.
